# The Last Word on the Enzyme Treatment of Cancer

**Published:** 1909-10-30

**Authors:** 


### SPECIAL ARTICLES.

### THE LAST WORD ON THE ENZYME TREATMENT OF CANCER.

The last word on the enzyme treatment of cancer has just been issued in the form of a scientific report from the Skin and Cancer Hospital of New York, where a committee has been investigating the subject during the last three years.

Dr. William Bainbridge has been the leading spirit of the investigation upon which this report is founded and it is published under his editorship.

Our readers will remember with what a " boom " the method of treatment under discussion was introduced to the notice of the profession. In the words of Dr. Bainbridge: " Through the over-zealous influence of certain medical writers for the lay press, and a few premature enthusiasts within the ranks of the profession itself, the method was heralded far and wide, and the patients soon began to make the demand that it be tried in their case."

In view of these facts, the staff of the New York Skin and Cancer Hospital determined to utilise the large amount of clinical material at their disposal in giving an extended trial to the enzyme treatment. No one who has the opportunity of reading their report can deny that the test to which they subjected the method was thorough and scientific.

A very large number of cases were dealt with, the majority suffering from incurable and inoperable growths of various kinds; a few, however, had early cancer quite amenable to surgical intervention, but were unwilling to undergo operative treatment.

Evidence of the completeness of detail with which the inquiry was carried out is furnished by the fact that in many cases when patients were unwilling or unable to remain in the wards of the hospital, medical attention and skilled nursing was provided by means of a special fund raised for the purpose in order that treatment and scientific observations might be continued in the patients' own homes, which in many cases were at a considerable distance from the hospital.

Further, during the whole course of these prolonged experiments the investigators kept in touch with Dr. Beard—the originator of the treatment—in order that they might be informed without delay of any modification of his original views as to the best means of carrying out the treatment.

Every case was dealt with upon a systematised plan, which included physical examination of a most painstaking nature, daily records of the progress of symptoms, weekly examinations of the blood and urine, and, where possible, the blood-pressure and weight. The oral and hypodermic administration of holadin, trypsin, and amylopsin was carried out according to the directions of Dr. Beard in every detail.

In some of the cases, however, the doses administered were much greater than those originally employed, and as much as 100 minims of the " quadruple X " solution was injected daily without untoward result—a striking commentary on the value of the records of " cures " and of " terrific " systemic results following the use of quite small doses, which were so plentiful in the medical and lay press two years ago.

In order to ascertain to what extent the trypsin treatment had been adopted by the profession generally, Messrs. Fairchild—the manufacturers of the preparations—were asked to furnish a list of all those to whom trypsin had been sold. To every doctor on this list a printed series of questions was sent, asking to what extent the enzyme preparations had been used and with what results.

Replies were received in 780 instances, furnishing accounts of 949 cases that had undergone the treatment. In 686 of these the results were negative, and in 248 the patients were said to have been favourably influenced as regards pain and general condition, but not as to the final result of the disease. Of the remaining 13 cases, some were still under treatment and in others apparent cure was claimed; but in some of them the diagnosis rested upon clinical evidence alone. In fact, in not one case of microscopically diagnosed cancer was there indubitable cure.

It is interesting that in the majority of the supposed cures the growth was in the stomach—a situation in which the clinical diagnosis is fraught with the greatest difficulty. Further, the strict regimen with regard to diet, etc., which was a necessary part of the treatment, possibly in large part explains the temporary general improvement met with in so many of the cases.

One hundred cases form the basis of the report under consideration. All were put under regular treatment and followed a strict régime. In a few cases, which were used as controls, injections of sterile water were substituted for the trypsin, and it is recorded that there was no difference in the rate of progress of the two classes of case. These 100 cases are arranged in tabular form and described with great minuteness; the nature of the growth being determined in nearly every case by histological examination and the progress and ultimate fate of the patient set out in detail.

The painstaking care with which these investigations appear to have been carried out and the wealth of detail furnished in the tables, and particularly the moderation with which the results are stated, cannot fail to convince everyone of the unbiased attitude and the judicial summing up of Dr. Bainbridge and those associated with him in what is a very valuable piece of research.

In all instances where it was possible, small fragments of the growth were from time to time examined by skilled microscopists, in order to determine the presence or absence of cellular changes which might be attributed to the treatment. Dr. Mallory—the distinguished pathologist at Harvard Medical School—found nothing that he could in any way attribute to the action of trypsin, and Dr. Martha Wollstein made a similar statement. Dr. James Ewing found degenerative changes in some of the specimens, but the changes were confined to the centre of the growth, which in each case was undergoing active extension at the periphery. The report terminates by giving a list of conclusions at which those responsible for the experiment arrived, and we cannot do better than quote verbatim some of the more important of these:—

1. That lotio pancreatis applied locally clears the ulcerating surface by removing organisms.

2. That control cases given injections of water, plus the régime, did as well as those on the full enzyme treatment.

3. That injectio trypsini in some cases appears to cause more rapid disintegration of cancerous tissue.

4. That while it may accelerate the breaking down of the centre, the periphery of the growth is found to be actively growing.

5. That because of the tendency of the trypsin to disintegrate tissues, it may be a direct menace to life either by eroding large blood-vessels or by overwhelming the system with toxic products.

6. That the injections are often so painful that the patients refuse to take them.

7. That the enzyme treatment as administered in the cases reported upon, and according to the suggestions of Dr. Beard, does not check the cancerous process.

8. That it does not prevent metastasis.

9. That IT DOES NOT CURE CANCER.

Although the trypsin treatment, in spite of much advertising, has never had such a vogue in this country as it has in America, and although the claims of those who were responsible for its introduction have been disproved by the extended series of observations carried out in the cancer wards of the Middlesex Hospital and elsewhere, it is very satisfactory that this additional and very convincing series of cases should have been brought forward to refute the statements of those few whose voices are still crying out in a very wilderness of unbelief.

